# Changing Behaviour: Blindness to Risk and a Critique of Tobacco Control Policy in China—A Qualitative Study

**DOI:** 10.3390/children9091412

**Published:** 2022-09-17

**Authors:** Tong Pei, Tingzhong Yang

**Affiliations:** 1 School of Humanities and Management, Zhejing Chinese Medical University, Hangzhou 310053, China; 2 Women’s Hospital/Center for Tobacco Control Research, Zhejiang University School of Medicine, Hangzhou 310030, China

**Keywords:** young adults, smoking, risk behaviour, tobacco control

## Abstract

(1) Background: It is well recognised that a focus on changing behaviour remains a dominant and often appealing approach to develop health policies. This study provides a sociological insight into young adults’ knowledge of the health effects of smoking cigarettes. We also examine the challenges in tobacco control and criticize the implementation policies in Chinese context. (2) Methods: The study applies both a micro-sociological and a macro-sociological approach using semi-structured interviews and documents as the primary research methodology. Fieldwork was conducted from July to September 2016 and December 2016 to March 2017. The qualitative study involved 45 semi-structured interviews with young adults aged 16–24 years (15 females and 30 males) in Tianjin, China. A grounded theory approach was used for a thematic analysis. (3) Results: The participants knew cigarettes are harmful, although they lacked a comprehensive understanding of the health risks of smoking. Because the health consequences usually emerge after a long period of smoking, young smokers decide to take the health risk. All participants have a general understanding of China’s tobacco control policies and think that the implementation is ineffective. (4) Conclusions: Changing in smoking is a process embedded in complex social environments and cultures. Smoking behaviour is not only a personal choice, but also related to personal connections with peers and identity in Chinese society. The Chinese government has made significant achievements in tobacco control since joining the WHO framework convention on tobacco control in 2005. However, implementation needs to be stricter in order to achieve international levels of control, especially in taxes on tobacco product and the price of cigarettes. There is an urgent need for the regulation of e-cigarettes in China.

## 1. Introduction

With a population of 1.4 billion, China has more than 300 million smokers (nearly one-third of the world’s total) and 740 million people are exposed to second-hand smoke, resulting in more than one million tobacco-related deaths annually [1,2]. If current trends continue, China’s annual tobacco-related deaths will reach 2 million by 2030 and 3 million by 2050 [3].

In addition to high rates of tobacco use among adults, the current tobacco smoking remains high among young adults aged 15–24 (17.9% in 2010 to 18.6% in 2018) [4]. Research has demonstrated that smoking in adolescence predicts smoking during adulthood (early and late 20 s) [5,6,7]. Data shows that 82.3% of students aged 13–15 years had first tried smoking by the age of 13 [8]. More than half of daily smokers aged 20–34 years old started daily smoking before the age of 20 [1]. 

Adolescence is in the crucial life stage for preventing tobacco initiation and its consequences because “this is the time in life (roughly ages 10 through 18 years) when onset, regular use and dependence begin” [9] (p. 543). From a public health standpoint, the detrimental impact of smoking on physical health and well-being has been widely documented since the 1960s [10,11,12]. For young people, the health consequences of smoking can be both short-term and long-term [9,13].

Preventing smoking among young people is also critical for ending the smoking epidemic. Much of the published research has discussed the factors associated with youth smoking use from quantitative levels, including social and physical environments (mass media, peer influence, and parents’ smoking behaviour), biological and genetic factors (young people may be sensitive to nicotine and teens can become dependent on nicotine sooner than adults), mental health (there is a strong relationship between youth smoking and depression, anxiety, and stress), and personal behaviour (lower income and education; lack of support from parents; poor study performance in school; and low self-image or self-esteem) [14,15,16,17,18,19,20,21,22]. However, we currently understand surprisingly little about how young people themselves think about why they do or do not smoke and how their understandings of risk behaviour should best be interpreted, especially in Chinese context. 

Denormalising smoking activities through policy interventions and social-marketing campaigns (e.g., smoking-free policies and campaigns; restriction on marketing) have played an important role in tobacco control. The first global health treaty, the World Health Organization’s Framework Convention on Tobacco Control (WHO FCTC) has provided a new legal dimension to international health cooperation by balancing tobacco demand reduction strategies with supply strategies [23]. Since China became a party to the WHO FCTC in 2006, social mobilisation and legislative responses to tobacco control have made substantial progress, including growing public awareness of the health hazards of smoking, legalising smoking-free laws in many cities and increasing the price of cigarettes [24]. Although tobacco control is improving in China, because of the large population of smokers (especially men, who are more resistant to change), tobacco control in China is still a great public health challenge.

Many studies have discussed implementing tobacco control policies in China based on the WHO FCTC [24,25,26], few studies look at this issue at the individual level by asking people what they know and how they think about the implementation of tobacco control interventions. Furthermore, few studies have discussed how these tobacco control strategies affect the understanding and behaviour of smoking among young adult smokers and non-smokers.

The aim of this study is to look at young adults’ smoking behaviour and perceptions of tobacco control policy. In this study, drawing on qualitative data, we first describe young adults’ knowledge of the health effects of smoking cigarettes. We then go on to explore young adults’ understanding of e-cigarettes and the experience of using them. Finally, we examine the challenges in tobacco control and criticize the implementation policies by interviewing young adults’ perceptions. The findings are relevant to the design of health promotion approaches targeting young smokers.

## 2. Materials and Methods

### 2.1. Research Methods

In contrast to the substantial body of quantitative research examining the factors that influence smoking behaviour among young people, this qualitative study focuses on young adults’ perceptions of smoking and tobacco control. The multiple sources of data, including interviews, documents and photographs, were used to increase the credibility of the research findings.

### 2.2. Data Collection

The fieldwork was conducted in Tianjin, China and was investigated in two stages: July to September 2016 and December 2016 to March 2017. Participants were recruited by using network and snowball sampling, which is often used in qualitative studies [27,28]. By using the network sampling method, we asked friends and relatives to approach potential participants. Most of them were quick to respond and tried their best to help us. This matches the argument that in the Chinese context, people who were well known to a researcher were more likely to participate in his/her research than others [29]. No matter whether in traditional or contemporary Chinese areas, strong reliance on interpersonal relations is the basis for social behaviour [30]. By using the snowball sampling method, we approached the first research participants, who is a daily smoker aged 18, and asked him to assist us in identifying other potential interviewees.

The selection of the participants was guided by age: young people aged 16–24 years (This age group has the largest increase in the smoking population, compared with other groups in China) who were resident in Tianjin, China, were asked to participate, whether or not they were smokers. Since issues of gender, education level, parental background and smoking status are of interest, a purposive sampling technique was applied to facilitate sample variety. In the end, 45 participants aged 16–24 years, with 15 female and 30 males were recruited. This study was submitted to, and approved by, the University of Essex Ethics Committee. The demographic characteristics of the 45 interview participants are summarised in Table 1. Smoking status of interviewees are shown in Table 2.

All interview questions were developed in English, translated into and conducted in Chinese, and then translated back into English for analysis (See Table A1). Seven months of fieldwork produced rich data with more than 800 pages of transcribed interviews and field notes. All the data were imported into the NVivo 10 software to facilitate analysis. All interviews were transcribed verbatim. After transcribing the data, the next step was to code and analyse the data. Grounded theory was used for data analysis.

## 3. Results

### 3.1. Knowledge of the Health Effects of Smoking Cigarettes

Non-smokers often choose to abstain from smoking because of health concerns. For instance, Qian, a male non-smoker said: “I believe that smoking has a big impact on health. First, it is definitely bad for the lungs. Sometimes I am exposed to second-hand smoke for a while and my throat does feel uncomfortable” (17-year-old, male, high school student, never-smoker).

Participants were aware of the risks of smoking but had limited knowledge of its health effects. They knew that smoking is harmful to health and can led to lung cancer, but they did not have a comprehensive knowledge of other diseases caused by smoking, such as chronic obstructive pulmonary disease (COPD), heart disease, and strokes. More importantly, knowledge of the dangers of smoking does not appear to have a great impact on young people’s smoking behaviour. In other words, although young smokers know the negative impact of smoking on health, they do not stop smoking. The following accounts are some of the examples:

*Wang: I know smoking causes lung cancer and makes teeth yellow. Smoking during pregnancy can have a bad effect on babies. But this information does not stop me from smoking. I just gain some knowledge. That’s it (22-year-old, male, undergraduate, occasional smoker).*

*Wu: I already knew smoking is bad for health when I was young. There is a health warning statement on the pack, saying smoking is bad for health. But it does not have any impact on me. Otherwise, I wouldn’t be a smoker now (18-year-old, male, high school student, occasional smoker).*

*Shi: Sometimes I think that those kinds of education campaigns are exaggerated to frighten smokers (22-year-old, male, undergraduate, occasional smoker).*

*Yue: I know smoking is bad for your health. But I don’t care much about it. As far as symptoms go, sometimes I smoke heavily then have a sore throat (24-year-old, male, employee, daily smoker).*

*Ying: Smoking is of course bad for health, and I obviously feel that my throat is always flawed. I only stop smoking when I am not feeling well, but I will start smoking again after I am in good health (23-year-old, female, employee, occasional smoker).*

*Shi San: Smoking certainly has an impact on health, such as heart and lungs. I care about my health, so I do some aerobic exercise to strengthen my heart and lungs (20-year-old, female, unemployed, daily smoker).*

As presented in the above accounts, young smokers recognized the contradictory kind of benefits that smoking provided. Young smokers were aware about the smoking habit damage to their health, such as ‘I know smoking is bad for health’. However, the knowledge of the health effects of smoking cigarettes did not stop them smoking. Hence, there must be other things in life got in the way of them acting on this and giving up smoking, for instance, peer pressure, stress relief and social networks. 

Kelly and Barker [31] criticize some common errors made in attempts to change health-related behaviour. One error is the belief that knowledge and information are the main drive of people’s behaviour. For example, many public health practitioners or behavioural science experts believe that if they tell people the negative consequences of smoking, they will change their behaviour accordingly. In reality, giving people information does not necessarily make them change their behaviour because it is strongly socially and culturally shaped. Michie [32] argues that as it is located in complex social environments and cultures, human behaviour is the result of the interplay between habits, automatic responses to the environments, conscious choice and calculation. 

Behaviours tend to be functional for people [31]. Smoking is both addictive and risk-taking behaviour. The negative consequences of smoking, such as increased mortality, do not show up immediately. The below accounts from two daily smokers proved that smokers prefer to enjoy the current pleasure from smoking rather than think about the long-term health harms.

*Wang Wei: I am not worried about health too much. That is the thing for the future. Sometimes I am thinking I have already smoked for several years, my lung has already become black, and it is meaningless to stop smoking now (24-year-old, male, employee, daily smoker).*

*Shen: Now I am young, I never think about the bad effect on my health. The health outcomes will appear when I am around 40 years old when I have smoked for a long time. When I am that age, I will think about stop smoking (18-year-old, male, migrant worker, daily smoker).*

Kelly and Barker [31] argue that another common error made in trying to change health-related behaviour is the idea that ‘people act rationally’. In reality, people often act irrationally. Smoking involves processes and practices ingrained in social life. Smoking is embedded in people’s everyday lives, their routines and habits. Self-identity is derived from this activity. This behaviour, to an important extent, helps people define who they are. Even when people accept the health information about the risks of smoking, behaviour change can be very difficult. As Qiu said:

*No matter how popular education is, whatever health warnings or ugly pictures on the packs, it is hard to change behaviour There are many education campaigns on TV or the Internet about smoking leading to lung cancer. Most smokers definitely know smoking is bad for their health. But when they are addicted, it is hard for them to stop smoking (22-year-old, male, undergraduate, daily smoker).*

Many scholars and campaigners claim that it is necessary to have much more propaganda to let young people know the health risks of smoking and establish a strong social norm against teenagers smoking. Globally, there are youth access policies to restrict young people’s access to cigarettes, including laws setting a minimum age for buying tobacco products, licensing tobacco retailers, penalties for selling tobacco products to minors, and limits on vending machine placements [14,33,34]. The assumption of these programmes is that restricting young people’s access will stop them from starting to smoke and disrupt the supply of cigarettes to underage smokers. However, there are controversies about restricting youth access. One key controversy is that it is unclear whether restrictions on youth access to cigarettes and education programmes can really stop underage smokers from getting cigarettes or even encourage them to smoke by making smoking seem adult and act of rebellion [32,35]. If young people perceive themselves as ‘rebels’ or ‘outside the mainstream’, restrictions on access and health education can make smoking seem more appealing as a symbol of rebellion against authority or adulthood [35,36]. 

In this study, interviewees expressed their opinions on this argument. Young smokers said that young people are often rebellious, so smoking interventions targeting young people could lead them to be much more curious about cigarettes or even are more likely to start smoking. The following accounts are some of the examples:

*Jinze: I don’t think there should be more education campaigns on the dangers of smoking among teenagers. Teenagers are rebellious. The more control government or teachers attempt, the more rebellious young people are (24-year-old, male, employee, daily smoker).*

*Jiang: It is hard to control smoking among young people. They are in adolescence and rebellious. They don’t even listen to their parents, let alone the public health campaigns (18-year-old, male, migrant worker, daily smoker).*

*Jiao: The education campaigns on the dangers of smoking should start from primary school. If it starts from junior or high school, the young people are in transition to adulthood and rebellious. The more you tell them not to smoke, the more like they will (22-year-old, male, teacher in college, daily smoker).*

Moreover, facing the health risk of smoking, smokers chose so-called ‘low-tar’ cigarettes because they believe these types of cigarettes expose them to less tar and are less harmful to their health than regular cigarettes.

*Liu: I suggest the tobacco industry should decrease the tar content in order to reduce the harm to smokers’ health (23-year-old, male, employee, daily smoker).*

*Jinze: Tar in cigarettes is the most harmful ingredient. The higher purity of tar cigarettes contain, the more harm it causes to smokers (24-year-old, male, employee, daily smoker).*

Studies have shown that the ‘Premiumization’ strategy to promote the concept that the premium brands of cigarettes are higher quality and less harmful used by the China National Tobacco Corporation (CNTC) does in fact work [37,38]. According to the accounts above, it appears that CNTC’s promotion strategy has an influence on people’s cigarette brand consumption choice. People have been wrongly led to believe that premium cigarettes are of better quality and less harmful. In fact, there is no scientific evidence shows that ‘low-tar’ light cigarettes are safer than regular cigarettes [39].

### 3.2. E-Cigarettes: A Healthier Alternative to Cigarette Smoking?

E-cigarettes arguably pose fewer health risks than smoking cigarettes, increasing smoking cessation and reducing the widespread harms of smoke tobacco [40,41]. They are often marketed by manufacturers with claims that they are a healthier alternative to cigarette smoking, that there is no second-hand smoke exposure, and that they help with smoking cessation [42,43]. Although evidence of these benefits remains contested and the long-term effects are unclear, many smokers are motived to use e-cigarettes to attempt to stop smoking [42,43,44,45,46,47,48]. 

China is a global production and export centre of the e-cigarette industry. Around 95% of the world’s e-cigarettes are manufactured in China, mainly in Shenzhen city [49]. Despite the high level of production, consumption of e-cigarettes in China is relatively low. This section discusses young adults’ reasons and experiences of e-cigarettes use. The attraction of e-cigarettes for non-smokers is also explored.

In the interview, no non-smokers expressed interest in e-cigarettes use. They even have limited knowledge about the product. All young participants who expressed interest in them or had ever tried them were cigarette smokers. The main reason these young adults give for vaping was to help them stop smoking.

*Ge: Some people cannot stop smoking cigarettes; they use e-cigarettes for cessation (22-year-old, male, undergraduate, occasional smoker).*

*Hao Wang: I used an e-cigarette for a period of time. In the beginning, I was curious about how e-cigarette helped stop smoking. I saw the advertisement on TV then bought one (24-year-old, make, employee, daily smoker).*

Apart from wanting to stop smoking, young people can be motivated to try to use e-cigarettes for fun and out of curiosity. The following accounts are some of the examples:

*Zheng: I tried an e-cigarette once out of curiosity (18-year-old, male, high school student, daily smoker).*

*Jiao: In 2014, e-cigarettes are very popular. My flatmate bought one and gave it to me to have a try. I tried it and I found it interesting and then I bought one for myself (22-year-old, male, administrator in college, daily smoker).*

*Jinze: Some people may use e-cigarettes to be special. For example, in the pub, people vape and there is lots of smoke. Vaping might make young people who never smoke want to try e-cigarettes. Young people are curious. They may try e-cigarettes if their peers smoke (24-year-old, male, employee, daily smoker).*

*Qiu: I have an e-cigarette. I heard about this product from my friend. I tried his e-cigarette once and it seemed interesting, then I bought one online for around RMB 400 (22-year-old, male, undergraduate, daily smoker).*

Not all e-cigarette users like the flavour nor have a pleasant experience using them. Participants complained that an e-cigarette was heavy to carry and needed to be recharged frequently. They did not use e-cigarettes often. None of them stopped smoking completely by using e-cigarettes. This finding is different from other studies which show that young people are attracted by flavoured e-cigarettes [46,50,51].

*Hao Wang: I used my e-cigarette for a month, but it did not help me stop smoking at all…. There is no nicotine in it and I still have a craving for cigarettes. Moreover, it was inconvenient to carry my e-cigarette, as it was heavy and needed to be recharged often. Compared to e-cigarettes, conventional cigarettes can be bought in the store easily (24-year-old, male, employee, daily smoker).*

*Jiao: I think most of the people who have smoked for a long time want to use e-cigarettes for stopping smoking. However, the refill liquids contain nicotine and can be added to e-cigarettes as much as you like. So vaping does not decrease the dose of nicotine compared to conventional cigarettes. Moreover, an e-cigarette is heavy to carry and not easy to use. This product especially does not work for elderly people, who have smoked cigarettes for ages and do not want to try a new product (22-year-old, male, administrator in college, daily smoker).*

*Yue: I bought an e-cigarette for smoking cessation and used it for a period of time, but it was not helpful at all. I felt I like I was inhaling air rather than smoke (24-year-old, male, employee, daily-smoker).*

*Shi San: I used an e-cigarette to stop smoking. But the fruit flavour was so disgusting that I did not use it anymore (20-year-old, female, unemployed, daily smoker).*

*Yang Wang: I used an e-cigarette for smoking cessation but it was not helpful. As an addicted smoker, I smoked cigarettes to wake myself up. But the e-cigarette did not give the same exact sensation as a cigarette, as there was no nicotine in it (24-year-old, male, employee, daily smoker).*

*Wang: I used an e-cigarette which was chocolate flavour and peach flavour…… A bad feature of e-cigarettes is that they have a plastic smell which disgusts me (22-year-old, male, undergraduate, occasional smoker).*

In the interview, e-cigarette users said that they used e-cigarettes to replace conventional cigarettes when they were not allowed to smoke conventional cigarettes in public indoors. For instance, Jinze, who is a daily smoker, said that the initial motivation for him to try an e-cigarette is replacing conventional cigarette smoking at home:

*I use an e-cigarette when I cannot smoke cigarettes, not for stopping smoking. …. My parents do not allow me to smoke when I am at home, so I smoke an e-cigarette to reduce my craving for cigarettes. …. A year ago, I saw e-cigarettes online. I spent over 500 RMB to buy an e-cigarette. I smoked it in front of friends to show I was vaping. I also introduced flavours to them for fun. Actually, I never think of stopping smoking. I was lying to my parents when I told them I smoked an e-cigarette to stop smoking because they did not want me to smoke cigarettes. But I was addicted to nicotine, especially when playing video games. The withdrawal feeling was really horrible. So I found e-cigarettes online, which I used at home (24-year-old, male, employee, daily smoker).*

It should be noted that many countries, including the UK, outlaw the use of e-cigarettes in public places. However, China does not have a national ban on e-cigarettes in public places, although the Beijing Tobacco Control Association has planned to promote the introduction of e-cigarettes into its tobacco control policies [52,53]. In Guangdong province, it is not permissible to smoke on a train or in a no-smoking area of trains or stations and this includes e-cigarettes. However, in most places of China where smoking cigarettes is banned and smokers want a nicotine hit, they may use e-cigarettes. 

Several empirical studies conducted in Chinese young people and adults, investigate the awareness and current use of e-cigarettes [52,53,54,55,56]. Results show that e-cigarettes were widely known and popular among Chinese adolescents aged between 12 and 18. There was a significant association between e-cigarette use and smoking cessation behaviour. Chinese adults were aware of e-cigarettes, while use was relatively low and most current users also smoked tobacco. Among smoking-related factors, cigarette smoking (ever and current), use of other tobacco products, second hand smoke exposure, and previous attempts to quit smoking were significantly associated with higher current e-cigarettes use in adolescents [56].

In this study, interviewees also talked about their understanding of the safety of e-cigarettes compared to conventional cigarettes. For instance, Liu believed that “vaping is less harmful than conventional cigarette smoking” (Liu, 23-year-old, male, employee, daily smoker). Participants thought e-cigarettes only contain nicotine, which is addictive yet does not cause cancer, but not tar. The following accounts are some of the examples:

*Jinze: Using e-cigarettes is a healthy hobby. E-cigarettes are not purely harmless, but vaping is much much less harmful than smoking…. Cigarettes have nicotine, which is addictive. Cigarettes also have tar and carbon monoxide, which are the unhealthy ingredients. But e-cigarettes do not have these unhealthy ingredients. Look at these liquid bottles, one says 6 mg/mL nicotine content and one is 3 mg/mL. At the beginning of using e-cigarettes, I used 12 and 6 mg/mL. After half a year, I decreased the nicotine content, from 12, 6 to 3 mg/mL. I did feel a big change in my body. After the first week of using it, I did not have phlegm anymore…. I checked online, which said that e-cigarettes are not good for the body but are not harmful either (24-year-old, male, employee, daily smoker).*

*Ge: E-cigarettes are less harmful than conventional cigarettes. However, generally speaking, both of them are bad for your health (22-year-old, male, undergraduate, occasional smoker).*

However, young smokers are more vigilant about e-cigarette ingredients and constituents. They do not have much information about exactly what they are and whether they are harmful. They believed e-cigarettes are at least as dangerous as smoking.

*Wang: I think e-cigarettes are harmful…. Users do not know what the liquid is (22-year-old, male, undergraduate, occasional smoker).*

*Jiao: I feel e-cigarettes are unsafe. The device has a battery. The liquid contains addictive ingredients, which maybe are less harmful than conventional cigarettes (22-year-old, male, administrator in college, daily smoker).*

*Wang Wei: I think an e-cigarette is much unhealthier than a conventional cigarette because the liquid is chemical rather than nicotine (24-year-old, male, employee, daily smoker).*

Studies show that smokers are often motivated to use e-cigarettes because they believe that they are less harmful than traditional cigarettes [56]. This awareness is often used by e-cigarette’ manufacturers to claim that there is no nicotine and only water vapour inside e-cigarettes. However, the fact is that the aerosols do contain nicotine and are a source of second-hand exposure to nicotine and other toxins [57,58,59]. According to a latest in vitro study published in Thorax, vaping damages immune cells in the lung, demonstrating that e-cigarettes are not 100% risk-free, although they are less harmful than ordinary cigarettes [60].

In recent years, there have been reports and news of e-cigarettes exploding and causing serious injuries [61,62]. Battery explosion is caused by faulty batteries or because the batteries were not handled as they should be. Another concern about e-cigarette is whether second-hand e-cigarette vapour is harmful. Although e-cigarettes do not create smoke like tobacco cigarettes, they do give off vapour that may contain harmful substances [63]. A latest study provides clinical evidence that vitamin E acetate most likely caused US vaping illness [64]. Another latest study finds that the level of some harmful components in e-cigarettes is even higher than in traditional cigarettes [65]. However, the scientific evidence on the harm of being exposed to second-hand e-cigarette vapour is still unknown. Hence, the harm of e-cigarettes should not be underestimated. It is important to explore people’s concerns about the safety of e-cigarettes and their experiences using them. 

### 3.3. How Do Tobacco Control Policies Influence Young People’s Smoking Behaviour?

#### 3.3.1. Smoke-Free Laws

When asking interviewees about their knowledge of tobacco control policies, smoking-free laws were mentioned the most. The term ‘smoke-free’ has been in use for decades to refer to stopping people smoking in public places and workplaces in order to protect others from exposure to second-hand tobacco smoke. Article 8 of the WHO FCTC makes it clear that country should provide protection “from exposure to tobacco smoke in indoor workplaces, on public transport, indoor public places and, as appropriate, other public places” [23] (p. 8).

In this study, all of the interviewees supported smoke-free bans and thought it was necessary to provide a smoke-free environment in public places. When talking about their own second-hand smoke exposure experiences, they were upset by environmental smoking exposure, especially in small developing cities or rural areas. Moreover, they rarely experienced any monitoring or fines for smokers who violated the bans. When talking about their opinions on the implementation of the smoking ban in public places, most of them said there were no-smoking signs in public places, yet complained that the enforcement did not work well. There were neither fines for smokers who smoked in public places (e.g., bars, restaurants, and indoor workplaces) nor any monitoring. The following accounts are some of the examples:

*Zhao: Many places have banned smoking, like taxis, the metro, hospitals, and shopping malls… But enforcement is bad. No fines, no monitors. Smokers still smoke in public places (17-year-old, female, high school student, never-smoker).*

*Man Ling: I heard there is a smoking ban in public places in Tianjin. You are not allowed to smoke in any public place with a ceiling. But the implementation does not work at all. There are no smoking signs on the wall but nobody pays any attention to them. There is no monitoring at all (20-year-old, female, undergraduate, never-smoker).*

*Ling Ge: No smoking signs are everywhere. But if customers smoke in restaurants, the business staff will not try to dissuade them from smoking (18-year-old, female, high school student, never-smoker).*

*Zheng: I feel the smoke-free ban is implemented well in Beijing. No smokers smoke in Beijing restaurants. But in Tianjin, smokers smoke in restaurants… I think if the fines are high enough, nobody would dare to smoke in public places (22-year-old, male, undergraduate, daily smoker).*

*Ge: I think the implementation of smoke-free law does not work at all. Non-smokers do not stop smokers who smoke in public places. Smokers just ignore no smoking signs in public places (22-year-old, male, undergraduate, occasional smoker).*

China does not yet have a comprehensive national smoke-free law; there are only some local-level regulations. The effectiveness of the implementation in different cities is different. The latest two smoking control ordinance in Beijing in 2015 and in Shanghai in 2017 are considered to be the strictest smoke-free legislation at the city level and are fully compliant with Article 8 in the WHO FCTC [24]. Under the rules, anyone who violates the bans must pay RMB 200 fines. The previous fine, seldom enforced, was just RMB 10. Anyone who breaks the law three times should be named on a government website, and businesses can be fined up to RMB 10,000 for failing to stop customers smoking on their premises. The ban prohibits smoking in all indoor public places and in places of business. Smoking rooms at airports were to be eliminated. After one year, a study evaluated the implementation of the 2015 Beijing ordinance and showed that the smoke-free environment around public places and primary and secondary schools was significantly better than one year before [66]. The best smoke-free places were hospitals and the most obvious improvement places were Internet rooms and café bars, although in public transport stations the ordinance was not fully implemented.

#### 3.3.2. Smoking Cessation Aids

The offer of smoking-cessation assistance is regarded as one of the fundamental WHO strategies to reduce global tobacco use [67]. The WHO FCTC recommends countries taking effective measures to promote cessation of tobacco use and adequate treatment for tobacco dependence, such as pharmaceutical products, counselling services on cessation of tobacco use and a telephone hotline [67]. In this study, among 19 daily smokers, 7 young people have more than 5 years smoking history and admitted that they were addicted to nicotine. Young smokers expressed that they have a weak awareness of smoking cessation aids and little experience in accessing any cessation approaches. For instance, Ge said:

*I do not trust any telephone quitline, clinics, whatever. I have never heard of it. I do not think the aid can really help me stop smoking. Stopping smoking is really a matter of using willpower (22-year-old, male, undergraduate, occasional smoker).*

An ex-smoker described his quitting experience:

*Pan: I started smoking when I was in high school. I was addicted to nicotine and cough often. When I was in college, I always wanted to quit but I could not. Until graduating from college, I really decided to stop smoking as I knew smoking is not good for me. Then I did it by willpower (21-year-old, male, undergraduate, ex-smoker).*

The discussion of the role of e-cigarettes as conventional cigarettes alternatives was presented earlier. Here, we focus on smoking-cessation clinics. In general, clinics play an important role in providing smoking-cessation services yet there are few in China. Wang et al. [68] conducted an investigation and analysis of the current status of smoking-cessation clinics in China in 2013. The results showed that the numbers of smoking-cessation clinics in operation in China decreased by 53%, from 201 in 2010 to 94 in 2013 [68]. Most clinics were located in developed cities, such as Beijing, Shanghai and Guangzhou province. Doctors said that it was difficult to maintain current smoking-cessation clinics because there were few outpatients. In Beijing, the awareness of smoking-cessation clinics was not high, 48% of smokers knew of this service and most of them (72.2%) had heard of the clinics from public campaigns [68]. The reasons why smokers did not go to clinics were they did not consider nicotine addiction a disease (46%) or they did not think clinics would be helpful (18%) [68].

Moreover, some medical personnel lack the awareness or capacity to provide cessation advice. For example, a study in Beijing showed that about one-third of the physicians did not receive training on smoking-cessation counselling [69]. Some other studies showed that physicians lack of interest to learn about tobacco and its health effects [70,71,72]. As a result, one-fifth of physicians never or seldom inquired about patients’ smoking status [70,73]. The reasons doctors gave for not asking patients if they smoked included beliefs that patients would not take their advice seriously and that smoking was irrelevant to the current illness [69,74]. Smokers also complained that the cessation programme was not available or affordable because the cost was excluded from Chinese health insurance. Another smoking-cessation service in China is a smoking telephone quitline. In 2013, the China Health Counselling Hotline began extending a cessation consultation service to ten cities in China. However, the use of the smoking telephone quitline is relatively infrequent [3]. 

#### 3.3.3. Cigarette Packaging

According to Article 11, the WHO FCTC recommends countries to implement plain (standardized) packaging of tobacco products and states that large and clear warnings should appear on both the front and back of cigarette packets [23]. Some studies have evaluated the effects of standardized packaging and found that graphic health warnings or pictures have a greater impact than words alone in increasing people’s awareness of the harms of smoking and reducing its appeal [75,76]. 

In this study, interviewees mentioned their attitudes towards the cigarette packaging of Chinese and foreign brands. They said that Chinese brands had only health warning sentences on the packs, while foreign brands of cigarettes were disgusting with ugly pictures on the packs. They did not buy such packs. However, neither health warnings nor ugly pictures make them want to stop smoking. The following accounts are some of the examples:

*Shen: I knew there was a health warning on the pack of cigarettes, saying ‘smoking is bad for health’, ‘stopping smoking is good for health’… I never buy the foreign brands that have ugly pictures on the packs. But most of the Chinese brands have delicate packages. Sometimes I would like to buy brands that have beautiful packages (18-year-old, male, migrant worker, daily smoker).*

*Ying: I know on the pack of Chinese brands of cigarettes there is a warning sentence saying ‘smoking is bad for health’. I know Thai brands of cigarettes have ugly pictures, which make me feel disgusted (23-year-old, female, employee, occasional smoker).*

*Shi San: The ugly picture on the pack of cigarettes definitely has an impact on me. I feel disgusted and I do not buy those brands of cigarettes (20-year-old, female, unemployed, daily smoker).*

**Figure 1:**
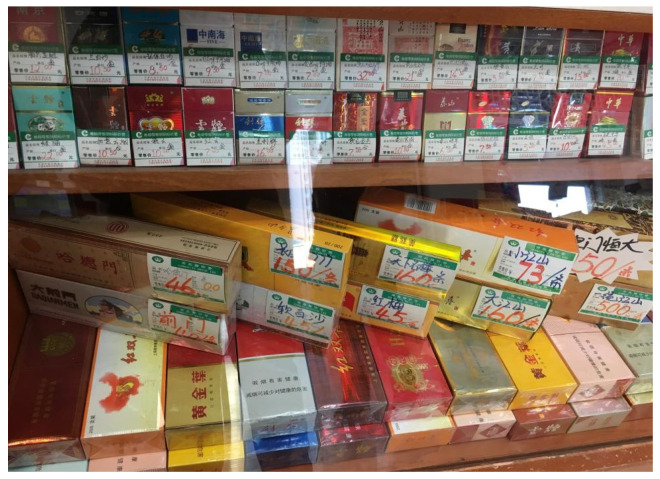
Display of cigarettes in a supermarket Tianjin, China on 17 January 2016.

Many countries, such as Australia, the UK, and Thailand, have introduced standardized packaging, which includes pictorial health warnings. In China, however, only text warnings (e.g., smoking causes cancer; smoking is harmful to your health) are included on cigarette packs. Moreover, in many countries, it is illegal to display tobacco products at the points of the sale in stores, although it is not in China (see Figure 1). Data shows that 64% of current Chinese smokers said that they were not influenced by the text warning packaging on Chinese brand cigarettes in their decisions about smoking [1]. State Tobacco Monopoly Administration (STMA) officials are strongly against the use of graphic warnings on packs and argue that this type of pack is incompatible with Chinese cultural traditions, such as gifting premium cigarettes [77].

#### 3.3.4. The Price of Cigarettes

In the fieldwork, smokers were asked about their attitudes towards the price of cigarettes in China. Most of them said that Chinese cigarettes were inexpensive, compared to foreign brands. The 2015 adjustment to tobacco taxation is the latest national tobacco tax reform in China [78]. Interviewees were asked their experience of the increased price of cigarettes. Young adults thought that although the price had increased, they still could afford to buy them. Because of the increase in the prices and the range in prices, some of them shifted to cheaper brands. No smokers said that they thought about stopping smoking because of the price increase. Here is what they said:

*Liu: Compared to some other countries, like Japan, the price of cigarettes in China is very cheap. Some brands of packs of cigarettes just cost RMB 2, which is too cheap (23-year-old, male, master graduate, daily smoker).*

*Shen: Last year, there was RMB 1–2 increase per pack. It has little effect. If the price increased by double, I would consider stopping smoking because it is too expensive… I think increasing the price may have more influence on young people as they do not work and have no income (18-year-old, male, migrant worker, daily smoker).*

*Gao Shou: Even though the price increased, I buy cheaper brands instead of stopping smoking (17-year-old, male, high school student, daily smoker).*

*Shi San: The price increased by like RMB 1 per pack. [Does it affect you?] More or less. I bought cheaper brands of cigarettes but did not decrease my consumption (20-year-old, female, unemployed, daily smoker).*

*Zhou: Although the price increased, let’s say from RMB 10 to RMB 20, there are still cheaper brands of cigarettes. Now, most people can afford the price of cigarettes and they do not care about the RMB 10 increase (18-year-old, male, high school student, daily smoker).*

*Jinze: I know the price increased by like 10%. I can afford it. Living expenses increased as well. I think this slight increase may influence young people much more because they have limited pocket money (24-year-old, male, employee, daily smoker).*

According to the accounts from smokers, they thought the price increase was too small and they still could afford to purchase cigarettes. Goodchild and Zheng’s study [79] assessed the impact of the 2015 Chinese tobacco tax increase on cigarette prices, sales volumes and the potential impact on smoking prevalence. They argue that although the price of cigarettes increased, cigarettes remain affordable in China and may become more so over time due to continued growth in people’s income. 

Increases in tobacco taxation have been widely regarded as one of the most effective measures for reducing tobacco consumption [67]. Higher taxation increases the retail prices for cigarettes, reducing their consumption, discouraging potential smokers from starting smoking, and encouraging smokers to stop smoking. This strategy is particular effective among young people. Since young people are among the most price-sensitive, high tobacco taxes makes cigarettes much less affordable for them. According to Article 6 of the WHO FCTC, the WHO recommends at least 70% of the retail price of cigarettes comes from excise tax, while the total tax rate would be about 51% at the retail price level in China [23,79]. Hence, taxes on tobacco products are not levied as much as is recommended. Compared to some developed countries, the price of cigarettes in China is still very low and the retail prices range variously from RMB 2.5–100 (£0.3–12) per pack [79].

## 4. Discussion

It is not easy to change smokers’ behaviour even when they have knowledge of health effects of smoking. It sometimes seems that public health policy is driven by the assumption that if we run campaigns using simple words or provide public information and knowledge, people will understand about the choice they have and then change to a healthier way of living. Sometimes we forget to understand the factors that lead to the behaviour in the first place. Looking back to understand human conduct and unravelling the causes of behaviour is likely to generate more effective and fundamental policies.

This study argue that it is not just individual behaviour which drives smoking epidemics; rather such behaviour takes place in social environments. Social, political and economic factors influence people’s health awareness and whether they change their behaviour to more healthy ways of living. Hence, efforts must take account of the social context and the political, economic and cultural forces which act directly on people’s health.

This paper also sought to understand why young adults use e-cigarettes. The findings revealed that there is little attraction to e-cigarettes for non-smokers. All interviewees who smoke had heard of e-cigarettes and some of them had tried e-cigarettes, while none of them continued to use e-cigarettes. Curiosity and the idea that they would help them to stop smoking are the two main reasons why they tried e-cigarettes. Reasons why they did not continue using them include dissatisfaction with products and safety concerns. With the rapid development of the e-cigarette market, China’s policy supervision on e-cigarettes is gradually strengthening, especially in the protection of minors. For example, it is forbidden to sell e-cigarettes to minors (including via the Internet). 

Based on opinion leaders for young people, this study argues that tobacco control policy should differ between adults and young people. The government needs to create intervention strategies targeting young people. Comprehensive smoking bans in schools need to be implemented strictly. It is also necessary for schools to set up tobacco control education courses and to make clear what the health risks are in detail and how to refuse the first cigarette. The misleading idea that smoking is a way to show masculinity, adulthood or to rebel needs to be changed. The state needs to pay attention to the negative impact of tobacco advertising targeted at young people. Parents, celebrities, and school teachers should set a good example for young people and avoid smoking in front of them. Traditional public health campaigns, such as preventing smoking uptake by young people, increasing their knowledge of the hazards of tobacco, and interventions with teachers and parents (e.g., smoke-free schools) remain important.

This study also examined the obstacles in the implementation of tobacco control in China by using both the bottom-up approach from the individual level and the top-down approach from political, economic and cultural levels. It has been argued that the significant factors that undermine China’s tobacco control are the high prevalence of smoking among men, the widespread social acceptability of male tobacco consumption, and the economic benefits to the state of tobacco production and sales. This study has explicated how these factors create difficulties in implementing tobacco control policy in China. 

Public policy-making process is embedded with the influences of the social context. International norms cannot be transferred into domestic laws unless they have been institutionalized at the national level. Social problems do not receive much attention from the state unless they are the most important and urgent. In terms of China’s tobacco control, there is a conflict and tension between pro-tobacco forces from the tobacco industry and the anti-tobacco forces from public health advocates. The WHO FCTC is a ‘soft’ international convention, which cannot be used to coerce its signatories to transfer FCTC policies into domestic laws. Whether or not the signatory states adequately implement the FCTC policies is determined by internal factors within states. In developed countries, the denormalization of smoking and low acceptability of smoking is conducive to enforcing effective tobacco control policies. However, in China the social context is not favourable for tobacco policy change. The economic profit from smoking is decreasing and the public cost of smoking is increasing. Hence, the government needs to pay much more attention to tobacco control, as it is an important public health strategy.

## 5. Limitations

One limitation of this study is that mainland China has a vast territory, cultural diversity, and large differences in economic and social development. The fieldwork was conducted only in Tianjin, a big city in the northern part of China. Hence, in a particular geographic context, the results cannot be generalized to the overall population of China. Further latest studies are needed in other regions, building on the important issues suggested by this study.

Furthermore, there could be a selection bias here, as participants were recruited via snowball sampling and social network. Smoking is not a healthy and desirable habit which the young smokers can talk proudly about to others, especially strangers. It is possible that those who had a particular interest in this topic were willing to participate in the discussion. In contrast, there may be individuals who were smokers under 18 or female smokers have felt more deterred to participant in the interview on this sensitive topic. It could therefore be that possibly relevant patterns of cigarette and e-cigarette users, which would have been substantial to explore the topic, are not reflected in the material, for instance, exclusive e-cigarette users. 

Another limitation may be the data timeliness. The data was collected between 2016–2017. Because of the fast development of e-cigarette market, the latest empirical studies are needed to examine online e-cigarette information exposure and its association with young adults’ e-cigarette use. More quantitative research is also needed for a comprehensive discussion.

## Figures and Tables

**Table 1:** The demographic characteristics of the 45 interview participants.

Demographic Categories	Number
Gender	
Male	30
Female	15
Age	
≤18	15
>18	30
State of current employment	
Unemployed	2
Employed	15
Student	28
Education	
Current education for students	
Academic high school	9
Vocational college school	3
Undergraduate	15
Postgraduate	1
The highest level of education for those who work	
Junior school	3
Vocational college	4
High school	3
Bachelor	6
Masters	1
Current smokers in the family excluding themselves	
Only father smoker	25
Only mother smoker	2
Both father and mother smokers	1
Sibling smokers	2
None	16

**Table 2:** Smoking status and history in interviewees.

Smoking Status ^1^		Total	Smoking History by Years
Non-smokers	Never-smokers	16	/
	Ex-occasional smokers	0	/
	Ex-smokers	1	6
Smokers	Daily smokers	19	3–11
	Occasional smokers	9	2–9
Total		45	/

^1^ Smoking status was verified using criteria based on the WHO’s guidelines for controlling and monitoring the tobacco epidemic. Available online: https://apps.who.int/iris/handle/10665/42049 (accessed on 10 September 2022).

## Data Availability

The data presented in this study are available upon reasonable request from the corresponding author. The data are not publicly available due because participants of this study did not agree for their data to be shared publicly.

## Appendix A

**Table A1:** Interview Guide.

*Topic 1: General information on smoking behaviours*		
		Could you tell me something about your smoking experience? Can you tell me about smoking in your family? What is the level of smoking in your peer group?
*Topic 2: Knowledge of the effects of tobacco smoke on health*		
		4. What do you know about the effects of tobacco smoke on health? 5. How did you get that information? 6. Did you know that smoking is harmful to non-smoker’s health? What effects have you heard about? 7. If you are a smoker, how long can you keep do not smoke? 8. Do you feel you are addicted to cigarettes? If so, have you ever planned on stopping? Why or why not? If you do not consider yourself addicted, why do you think so? 9. Have you ever tried stopping? If yes, why did you do so and how was the outcome? If not, why did not you do so? 10. Have you been advised to stop smoking by anyone (e.g., doctors; dentists; family members; friends; teachers)?
*Topic 3: Understanding of tobacco control policies*		
		11. What do you know about the tobacco control policy in China? 12. How did you learn about this? 13. Do you think the tobacco control policy in China is implemented effectively? Why? 14. To what extent do you think tobacco control policies influence young people’s smoking? 15. What type of anti-smoking campaign do you think is the most effective?
